# An interventional pilot of customized adherence enhancement combined with long-acting injectable antipsychotic medication (CAE-L) for poorly adherent patients with chronic psychotic disorder in Tanzania

**DOI:** 10.1186/s12888-022-03695-8

**Published:** 2022-01-27

**Authors:** Jessie Mbwambo, Sylvia Kaaya, Isaac Lema, Christopher J. Burant, Catherine Magwiza, Kim Madundo, Godwin Njiro, Carol E. Blixen, Kristin A. Cassidy, Jennifer B. Levin, Martha Sajatovic

**Affiliations:** 1 Department of Psychiatry and Mental Health, Muhimbili University of Health and Allied Sciences, School of Medicine, Dar es Salaam, Tanzania; 2 Frances Payne Bolton School of Nursing, Case Western Reserve University, Cleveland, OH USA; 3 Department of Psychiatry and Neurological & Behavioral Outcomes Center, Case Western Reserve University School of Medicine and University Hospitals Case Medical Center, Cleveland, OH USA; 4 Department of Psychiatry & of Neurology, Case Western Reserve University School of Medicine and University Hospitals Cleveland Medical Center, W.O. Walker Bldg, 7th Floor, 10524 Euclid Avenue, Cleveland, OH 44106 USA

**Keywords:** Schizophrenia, Treatment adherence, Antipsychotic, Psychosis

## Abstract

**Background:**

Chronic psychotic disorders (CPD) impose a particularly significant burden in resource-limited settings. Combining long-acting antipsychotic medication (LAI) with a customized adherence enhancement intervention (CAE-L) has potential to advance care.

**Methods:**

Nineteen adults ≥ age 18 with CPD who self-reported missing ≥20% of antipsychotic medication within the last month were stabilized on oral haloperidol prior to transitioning to monthly haloperidol decanote for 25 weeks. Outcome evaluations were conducted at baseline and Week 25. Primary outcomes were oral medication adherence assessed via the Tablet Routines Questionnaire (TRQ) and LAI injection frequency. Secondary outcomes included CPD symptoms measured by the Brief Psychiatric Rating Scale and Clinical Global Impressions, functioning evaluated using the Social and Occupational Functioning Scale, and medication attitudes assessed with the Drug Attitudes Inventory.

**Results:**

Mean sample age was 38.79 (SD = 9.31) with 18 individuals completing the study. There was one serious adverse event, a relapse into substance use, not deemed study-related. Mean endpoint LAI dosage was 65.79 mg (SD = 22.38). TRQ mean scores were 21.84 (SD =13.83) and 12.94 (SD = 11.93) at screen and baseline respectively. For only two individuals who were on concomitant oral medication at 25 weeks, TRQ change was not calculated. LAI injection frequency was 100%. Medication attitudes scores significantly improved from 7.89 (SD = 2.72) to 9.83 (SD = 0.52) (*p* = .001.) Changes in CPD symptoms and functioning were non-significant.

**Conclusions:**

CAE-L appears to be preliminarily feasible and acceptable in Tanzanians with CPD.

**Trial registration:**

The study was registered on ClinicalTrials.gov (NCT04327843) on March 31, 2020.

## Introduction

Chronic psychotic disorders (CPDs) occur world-wide but impose a particularly significant burden in resource-limited settings where the professional workforce is spread thin and access to both medication and behavioral treatments may be limited [1]. In Sub-Saharan Africa (SSA), as is the case for people with CPD in other parts of the world, poor medication adherence is seen in approximately half of individuals with CPD and is a major driver of relapse [2–6].

Reflecting the broad use of antipsychotic medications, a recent report from Namibia found that antipsychotic medications were the most widely consumed psychotropic medicines (84% were anti-psychotics) vs. 9.2% anti depressants and 6.8% anxiolytics [7]. Because a major impediment to adherence in CPD is difficulty with consistent medication routines, long-acting injectable antipsychotic medication (LAI) can be a potentially efficient and effective treatment option [8, 9]. An advantage for LAI is that can be administered monthly or even less frequently, eliminating the daily need to take medications which in itself can be a stigmatizing behavior [10]. But medication alone is unlikely to modify long-term attitudes and behaviors, and LAI is not a stand-alone care approach for CPD given the long-term and individual care needs of people with CPD [11, 12].

A brief, practical behavioral approach that maps onto individual patient reasons for poor adherence and which is intended to be used as a complement to LAI has been developed by a U.S. study team [13, 14]. To be feasible in lower-resource settings, effective interventions need to be able to be delivered by diverse types of staff, should be evidence-based, and should be able to be readily scaled-up.

Combining LAI with a customized adherence enhancement behavioral intervention (CAE-L) is an approach that has potential to advance care for people with CPD in resource-limited settings. This report describes a first-ever testing of CAE-L in poorly adherent patients with CPD in Tanzania.

## Materials and methods

### Overview

This as a 6-month prospective, non-controlled trial of CAE-L in 20 poorly adherent patients with CPD in Dar es Salaam, Tanzania. The study is part of a larger U.S. National Institute of Mental Health (NIMH)–funded trial described in greater detail elsewhere [15]. The LAI used in the study, haloperidol decanote, is widely available for the treatment of individuals with CPD in SSA [7, 16]. The behavioral intervention used in the study (Customized Adherence Enhancement/CAE) was delivered by social workers who were trained to follow a detailed curriculum. The study team used a mix of qualitative and quantitative evaluations to adapt the U.S. version of CAE to be culturally and linguistically appropriate for this setting [15]. Patient outcome assessments included adherence behaviors and attitudes, CPD symptoms, and functional status.

### The CAE-L intervention

The CAE-L intervention was previously tested in 2 U.S. studies involving patients with CPD. Patients received monthly CAE combined with LAI (CAE-L) for 6 months [14, 17]. In one of the U.S. studies, the LAI used was haloperidol decanoate, a first-generation LAI that is widely available in lower-resources settings [16, 17]. Drawn from iterative pilot work, CAE-L is flexibly delivered as a series of up to 4 treatment behavioral modules for which use is determined based upon an individual’s reasons for non-adherence (adherence barriers) [10, 14, 18, 19]. Adherence barriers are identified using 2 standardized measures, the Rating of Medication Influences (ROMI) and the Attitudes toward Mood Stabilizers Questionnaire (AMSQ) [20–22]. The 4 available modules are: 1) Psychoeducation focused on medication and consequences of missing medication; 2) Modified Motivational Enhancement Therapy (MET) to address non-adherence related to substance use; 3) Communication with Providers to facilitate appropriate treatment expectations and optimize management of feared or experienced side effects; 4) and Medication Routines intended to incorporate medication-taking into lifestyle. The modules are intended to be delivered by a clinical staff member during the same visit that the individual with CPD receives their LAI injection.

### Intervention site

The study setting where patients were enrolled was Muhimbili National Hospital, a 70-bed national referral hospital located in urban Dar es Salaam, Tanzania. It is the only psychiatric national referral center and serves a population of approximately 4.5 million. Patients are referred from 4 catchment zones that include 3 regional public and private hospitals.

### Study population

The sample comprised 19 adult patients ≥ age 18 with schizophrenia who self-reported missing 20% or more of antipsychotic medication within the last month, an established benchmark for poor adherence [23]. Adherence was measured using the Tablets Routines Questionnaire (TRQ) which measures medication adherence as a percentage of medication missed or skipped [24, 25]. Eligible patients had to agree to receive LAI and be able to participate in research activities. Written informed consent was obtained from all subjects/patients. Exclusion Criteria will include: 1.) Individuals on LAI immediately prior to enrollment, or those with intolerance or resistance to LAI; 2.) Medical conditions that would interfere with the patient’s ability to participate in the trial; 3.) Physical dependence on substances likely to lead to withdrawal reaction; 4.) Immediate risk of harm to self or others; and 5.) Pregnancy or lactation.

### LAI

Patients already on oral haloperidol were switched to haloperidol decanoate and stabilized. Individuals not on antipsychotic medication at the time of screening assessment or those on a different antipsychotic medication received an oral tolerance test (OTT) consisting of up to 14 days of oral haloperidol 4–10 mg/day. If the OTT suggested good tolerability, the participant then received LAI (haloperidol decanoate) intramuscularly (IM) after completion of baseline assessments. Dosing of LAI, given once every month for 6 months, was prescribed as clinically indicated by the treating research psychiatrist using conservative dosing to minimize drug-related adverse effects.

### Concomitant treatments

Stable dose oral psychotropic drugs (> 30 days of previous use) other than antipsychotics were continued. New psychotropic medication starts were not permitted. Medications for side effects could be given at the discretion of the treating research psychiatrist.

### Study measures

Baseline information included duration of psychiatric illness, past hospitalizations, past antipsychotic medication treatment history, and cumulative medical burden as evaluated by the self-reported Charlson Index [26]. Substance use was measured with the Alcohol Use Disorders Identification Test (AUDIT) and the Alcohol, Smoking and Substance Involvement Screening Test (ASSIST) [27, 28].

Outcome assessments were conducted at study baseline and at Week 25 follow-up (study endpoint). Similar to U.S. studies testing this blended LAI + behavioral intervention [13, 14] the primary outcomes were change in oral psychotropic medication adherence (for those who were also on oral drug at both study baseline and study endpoint) as measured by the TRQ and the mean LAI injection frequency. A participant was considered adherent if LAI was administered within 1 week of the time that it was originally scheduled to be administered. Secondary outcomes were change in adherence attitudes as measured by the 10-item Drug Attitude Inventory (DAI) and change in CPD symptoms as measured by the 18-item Brief Psychiatric Rating Scale (BPRS) and the single item Clinical Global Impressions (CGI) [29–31]. The BPRS version scoring ranged from 0 (no symptoms) to 7 (most severe). Change in functional status was evaluated using the Social and Occupational Functioning Scale (SOFAS) [32] .

### Safety/laboratory evaluations

Safety evaluations included basic laboratory evaluations (serum comprehensive metabolic panel, lipid profile, CBC with differential, and HIV as well as urine pregnancy testing for women) and EKG. Patient vital signs and weight were collected at each study visit. Standardized measures of extrapyramidal symptoms were assessed with Extrapyramidal Symptoms Scale-Abbreviated version (ESRS-A) [33]. Finally, reported side effects were evaluated at each study visit using a standardized format.

### Data analysis

As this study was mainly focused on feasibility, patient acceptability, and research capacity-building, we assessed only descriptive statistics and change from baseline in the primary and secondary measures using dependent samples paired t-tests to assess pre-post data in the sample that completed the study. This parametric technique was used, as there were no violations in the assumptions for the analyses (e.g., normality in the mean difference scores) and the techniques was robust to type 2 error. Data analysis was conducted using SPSS ver. 27 software.

## Results

### Study enrollment

**Fig. 1:**
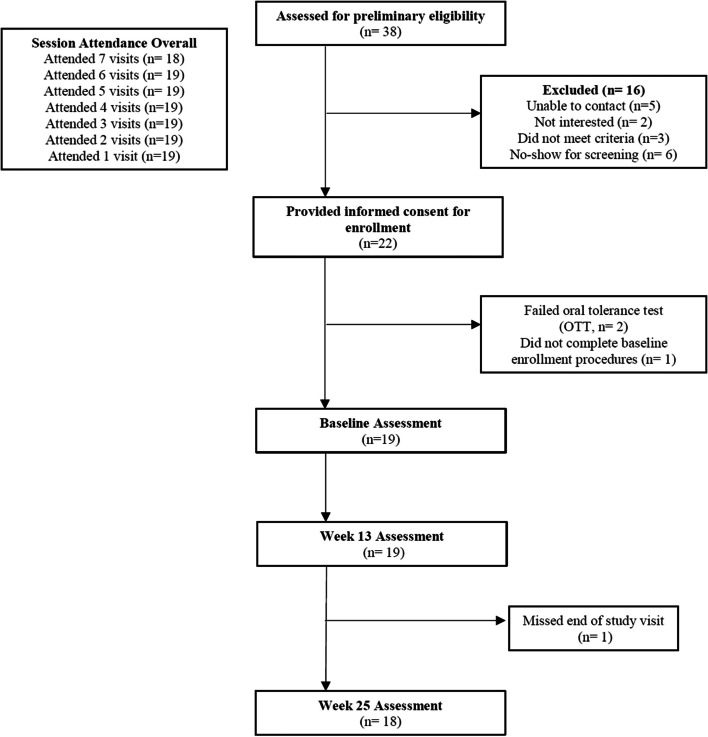
Study flow and enrollment

Figure 1 illustrates study flow and enrollment. Screening evaluations began on Oct 28, 2019 and the first participant was enrolled on Nov 5, 2019. There were 38 individuals who were initially approached for enrollment, with 22 individuals who appeared to fit preliminary study entry criteria and provided informed consent. Of these, 2 individuals were unable to tolerate OTT and did not proceed to study baseline. There were 19 individuals who completed baseline evaluations. Of these, all individuals participated in all study procedures and assessments except for one individual who missed the endpoint study assessment at week 25. All study participants were outpatients.

### Baseline sample

Table 1 shows the baseline study sample. Notably, this was a relatively young sample with a mean age of 38.79 (SD = 9.31), 73.7% male (*n* = 14) with a CPD duration of 18.89 (SD = 10.87) years. Most had multiple previous hospitalizations for CPD and had been treated with a variety of oral antipsychotic medications in the past. None had been on haloperidol decanoate in the past. As this was a clinically “stable” sample at baseline, mean total BPRS and CGI scores were relatively low.

**Table 1:** Baseline demographic information of enrolled participants (*N* = 19)

Variable	Mean (SD)	N (%)
**Age in years**	38.79 (9.31) range (27–64)	
**Sex**		
Male		14 (73.7%)
Female		5 (26.3%)
**Marital status**		
Single/never married		12 (63.2%)
Married		2 (10.5%)
Separated/divorced		4 (21.1%)
Widowed		1 (5.3%)
**Educational level in years**	8.37 (2.99) range (5–17)	
**Employment**		
Full-time		4 (21.1%)
Part-time		3 (15.8%)
Unemployed		12 (63.2%)
**Age of CPD onset in years**	19.89 (4.47) range (15–33)	
**Duration of CPD in years**	18.89 (10.87) range (4–47)	
**Number of previous hospitalizations**		
For CPD	6.16 (7.55) range (0–30)	
For substance abuse	0.05 (.23) range (0–1)	
Past physical abuse		6 (31.6%)
Past sexual abuse		1 (5.3%)
Family history mental illness		10 (52.6%)
Family history substance abuse		10 (52.6%)
**AUDIT score**	1.84 (3.13) range (0–12)	
**ASSIST score**	1.58 (2.65) range (0–10)	
**Self-reported Charlson Index Score**	0.37 (0.50) range (0–1)	
**Body Mass Index (BMI)**	22.70 (4.89) range (15.62–34.29)	
**Past oral medication history**		
**Chlorpromazine**		8 (42.1%)
**Fluphenizine**		11 (57.9%)
**Haloperidol**		14 (73.7%)
**Olanzapine**		3 (15.8%)
**Promethazine**		8 (42.1%)
**Risperidone**		5 (26.3%)
**Screening TRQ**^**a**^	21.84 (SD =13.83)	
**Baseline TRQ**^**a**^	12.94 (11.93) range (0–33)	

^a^Tablets Routine Questionnaire: self-reported proportion of missed oral medication in the last 30 days. Scores range from “0” (perfect adherence) to “100” (did not take any prescribed medication). Mean Tablet Routines Questionnaire calculated only for oral CPD medications (antipsychotics, mood stablizers, antidepressants). If more than 1 oral medication was prescribed. an average TRQ was calculated. Screening sample TRQ, N = 19, Baseline sample TRQ, N = 18

*CPD* chronic psychotic disorders

*AUDIT* Alcohol Use Disorders Identification Test

*ASSIST* Alcohol, Smoking, and Substance Involvement Screening Test

### Safety data and LAI dosage

Among the 19 individuals who completed baseline evaluation and went on to received CAE-L, there was 1 serious adverse event (SAE) in which 1 individual had a relapse into substance use and was breifly hospitalized. This SAE was not deemed to be related to this individual’s particiaption in the study. In addition, 13 participants had reported side effects at some point during the 25 week trial. All reported side effects were mild. There were only 2 participants who reported side effects present during the last visit. One individual reported blurry vision during the last visit which was not reported in the previous visits. Another individual reported mild muscle pain during that was present at baseline, resolved during most of the course of the trial, and then was reported again at study endpoint. Out of the 19 individuals who received medication treatment, the most common side effects were tremor (*N* = 7, 37%), drowsiness (*N* = 6. 32%) blurry vision (*N* = 3, 16%), restlessness (*N* = 2, 11%), and muscle pain (N = 2, 11%). The following were reported once: dry mouth, bad taste, nausea, twitching, headache, dry eyes and lightheadedness. No side effects were associated with study discontinuation. The mean endpoint dosage of haloperidol decanoate was 65.79 mg (SD = 22.38). There were no EKG or laboratory testing changes that were deemed to be clinically significant.

### CPD and other health outcomes

Table 2 shows group mean scores for the key outcomes of interest. Mean TRQ score, calculated only for oral medications, was 21.84 (SD =13.83) at screening. TRQ scores at baseline could only be calculated for 18 individuals and showed a mean of 12.94 (SD = 11.93). As only 2 individuals were on concommitten oral CPD mediation at 25 week follow up, mean change in TRQ was not calculated. LAI injection frequency was 100%. Mean baseline scores on BPRS and CGI decreased from 27.00 (SD = 10.26) to 25.06 (SD = 8.53) and 2.88 (SD = 1.32) to 2.24 (SD = 1.09) respectively at the 25-week follow-up, a non-significant change. Mean SOFAS score change was also non-significant. In contrast to their being no change in symptoms, total DAI score improved from 7.89 (SD = 2.72) at baseline to 9.83 (SD = 0.52), a significant improvement (*p* = .001).

**Table 2:** Change in key outcomes from baseline to 25 weeks

Variable (mean, SD)	Baseline Mean (SD)	25-weeks Mean (SD)	Statistic***
**BPRS**	**27.00 (10.26)**	**25.06 (8.53)**	***p*** **= .43**
**CGI (*n*** **= 17)**	**2.88 (1.32)**	**2.24 (1.09)**	***p*** **= .07**
**DAI**	**7.89 (2.72)**	**9.83 (0.52)**	***p*** **= .001**
**SOFAS**	**62.17 (18.28)**	**68.39 (15.28)**	***p*** **= .10**
**ESRS-A** **Parkinsonism**	**0.06 (.24)**	**0.06 (.24)**	***p*** **= 1.00**
**ESRS-A** **Dystonia**	**0.00 (.00)**	**0.00 (.00)**	***p*** **= NA**
**ESRS-A** **Dyskinesia**	**0.17 (.51)**	**0.11 (.47)**	***p*** **= .75**
**ESRS-A** **Akathisia**	**0.28 (.75)**	**0.00 (.00)**	***p*** **= .14**
**BMI**	**22.79 (5.02)**	**22.92 (5.58)**	***p*** **= .75**

***: pre/post comparison of baseline to endpoint means for 18 individuals who completed the 25-month trial

*BPRS* Brief Psychiatric Rating Scale

*CGI* Clinical Global Impression

*DAI* Drug Attitude Inventory

*SOFAS* Social and Occupational Assessmsent of Functioning Scale

*ESRS-A* Extrapyramidal Symptoms Scale-Abbreviated version

*BMI* Body Mass Index

ESRS scores were relatively unchanged from 0.06 at baseline and 0.06 at endpoint for Parkinsonism; 0.00 to 0.00 for Dystonia; 0.17 to 0.11; and 0.28 to 0.00 for Akathasia. BMI was also relatively unchanged. In addition to standardized measures of CPD and other health outcomes, we also assessed patient-perceived satisfaction with the behavioral component of the program. Among the individuals who provided satisfication with intervention input at 25 weeks, *N* = 18 (100%) strongly agreed or agreed that CAE was useful to them.

## Discussion

The purpose of the pilot trial was to determine the feasibility of providing combined treatment with a behavioral program intended to promote medication adherence combined with LAI (haloperidol decanoate) among poorly adherent Tanzanian with CPD. Based on this preliminary experience, the CAE-L intervention appears to be feasible and has high acceptability among patients. As the enrolled sample had minimal psychotic symptoms at baseline, there was minimal symptom improvement. There are several aspects of this pilot work that are worth noting which have implications for clinical care and future research planning.

Our sample appears to have similarities with other reports from SSA. A recent review of treatments for schizophrenia in SSA by Chidarakire and colleagues highlighted the limited mental health services related to financial constraints, lack of qualified mental health professionals, and problems in care access [34]. In this review, the majority of people with schizophrenia were treated with first-generation antipsychotics [35–37]. In our sample, the most common previous antipsychotic drugs were the first-generation antipsychotic drugs haloperidol, chlorpromazine, and promethazine. Individuals in our sample had generally extensive histories of previous relapse. A report by Kazadi et al. found that many patients with CPD discontinue their medication [36]. While our sample was, a priori, confined to individuals known to be poorly adherent, this is a sizable chunk of the CPD population and could be a useful program, especially among individuals who have had recent relapse due to poor adherence. Families in the review by Chidarakire reported a high level of burden associated with caring for a relative with CPD [34]. In our sample, we did not assess family distress, but found that approximately half of individuals with CPD also had a family psychiatric history, a factor that seems likely to add to overall family burden. Future work might include involving family members more extensively as part of patient recovery.

Of the 19 individuals who completed baseline assessments in the study reported here, 18 (95%) remained on the CAE-L intervention at 25-week follow-up. The LAI also appeared to be relatively well tolerated with only one SAE during the 6-month study period, a hospitalization that was due to substance use relapse and not deemed to be related to study participation. The most common side effects appeared to be related to extrapyramidal symptoms (tremor, muscle pain, restlessness), sedation and blurred vision, all known potential side effect of haloperidol. All side effects experienced by study participants were mild in severity. Previous studies support the notion that switching to LAI among individuals who are already known to readily tolerate oral haloperidol would not entail increased somatic burden or risk [38].

We saw improvement in self-reported medication treatment adherence between study screening and baseline time-points. This is consistent with other reports for this intervention and likely are due to Hawthorne effects in that observing adherence behavior appears to promote improved adherence, at least for the short term [39]. We did not see change in oral medication adherence from study baseline to the 25-week study endpoint. However, LAI adherence was 100% in this Tanzanian sample. It is also possible that change in self-reported oral medication adherence could have been obscured by the small number of individuals on oral medication at study endpoint. In this Tanzanian sample, individuals were, for the most part, entirely transitioned off of oral medications once LAI was stabilized/maintained. These findings are in contrast to the U.S. studies with the CAE-L intervention where patients continued to receive a variety of oral drugs in addition to LAI [13, 14]. Given the disproportionate burden of CPD in SSA, the fact that CAE-L participants in Tanzania no longer were on oral medication could have important clinical implications. For example, LAI non-adherence, if and when it occurs, is more readily detected and clinicians and families have the opportunity to intervene in a timely fashion.

Our study also did not find change in clinical symptoms, or functional status from study baseline to the 25-week study endpoint. It is possible that the minimal symptom change could have been due to the fact that individuals were already treated and stabilized on oral haloperidol prior to initiating LAI. Studies conducted in the U.S. using the CAE intervention with individuals who are more symptomatic at baseline suggests that global psychopathology and functional status has potential to improve [12, 14]. In contrast to the lack of change on symptoms and functioning, we saw significant improvement in attitudes towards medication, a finding that has also been seen in U.S. samples that have received the CAE-L intervention [13, 14]. Our study did not capture results of outcomes beyond the 6-month follow-up time point, however a potentially important domain to assess in future work is whether improved attitudes toward CPD medication treatment translates to longer-term adherence promotion and enhanced recovery.

A recently complete systematic literature (SLR) review of the global literature on antipsychotic medication trials in SSA found only 26 studies published from 1994 to 2019 [16]. Studies were from Nigeria (*n* = 3), South Africa (*n* = 21), Malawi (*n* = 1), and there was 1 multicenter study in Nigeria and South Africa. There were no studies from Tanzania. Like the study reported here, the majority of SLR sample participants were male. The primary adverse effects were changes in metabolic outcomes, a finding not seen in our report. The sample in the study reported here had a BMI considered to be within the normal/health range and lower than one typically sees in reports form the U.S. and other high income countries [40]. Also similar to the SLR, we also did not see significant changes in extrapyramidal symptom measures, possibly related to the fact that our study sample was relatively young (< age 39 years).

Despite the promising preliminary feasibility findings, this study has a number of limitations. The small sample size, single-site enrollment, and the fact that poor medication adherence was one of the inclusion criterion may make findings less generalizable to the full spectrum of patients with CPD in Tanzania. Additionally, since we did not collect data on family burden, it is not possible to make any conclusions regarding how CAE-L may impact families and communities. On the other hand, the fact that the study used resources that might be expected to be available in at least some low-resource settings (a first-generation LAI, social workers to deliver the behavioral intervention) may increase generalizability and suggest that future studies are warranted given the pervasiveness and corrosive effects of sub-optimal medication adherence among patients with CPD.

Finally, a key benefit to LAIs is the potential to reduce relapse and subsequent hospitalizations. Because our study was an NIH-funded 2-year project, intended to assess the feasibility and preliminary efficacy of the blended LAI and behavioral intervention, individual study participants were followed for a 6-month time-period. During this time period, there was only 1 individual who was hospitalized. The short duration of follow-up and small sample make it difficult to draw conclusions regarding potential overall impact on hospitalizations and our inability to provide information on hospitalization trajectory is a limitation of our report.

## Conclusion

Combining LAI with a customized adherence enhancement behavioral intervention (CAE-L) is an approach that has potential to advance care for people with CPD in resource-limited settings. While this first-ever testing of CAE-L in poorly adherent patients with CPD in Tanzania is promising, future work needs to include a larger sample and a comparison arm to more clearly determine whether this approach can yield additional benefits beyond current clinical practice.

## Data Availability

The data that support the findings of this study are available on request from the corresponding author (MS) to qualified individuals and with appropriate cross-institutional data use agreements. The data are not publicly available due to their containing information that could compromise the privacy of research participants.

## Abbreviations

CAE-L: Customized Adherence Enhancement with Long-acting injectable medication
NIMH: U.S. National Institute of Mental Health
CPD: Chronic Psychotic Disorders
SSA: Sub-Saharan Africa
LAI: Long-Acting Injectable antipsychotic medication
U.S.: United States
CAE: Customized Adherence Enhancement
ROMI: Rating Of Medication Influences
AMSQ: Attitudes toward Mood Stabilizers Questionnaire
MET: Modified Motivational Enhancement Therapy
TRQ: Tablet Routines Questionnaire
OTT: Oral Tolerance Test
IM: Intramuscularly
AUDIT: Alcohol Use Disorders Identification Test
ASSIST: Alcohol, Smoking and Substance Involvement Screening Test
DAI: Drug Attitude Inventory
BPRS: Brief Psychiatric Rating Scale
CGI: Clinical Global Impressions
SOFAS: Social and Occupational Functioning Scale
CBC: Complete Blood Count
HIV: Human Immunodeficiency Virus
EKG: Electrocardiogram
ESRS-A: Extrapyramidal Symptoms Scale-Abbreviated
SPSS: Statistical Package for the Social Sciences
SAE: Serious Adverse Event
BMI: Body Mass Index
SLR: Systematic Literature Review
